# Bioequivalence of tegomil fumarate gastro-resistant capsules versus dimethyl fumarate gastro-resistant capsules under different fed conditions: two randomized, crossover, phase I studies

**DOI:** 10.3389/fneur.2026.1936043

**Published:** 2026-09-29

**Authors:** Katja Zietz, Simona Rizea Savu, Lina Remis, Natalia Nicolau, Adriana Iordachescu, Florian Herb

**Affiliations:** 1 Alfred E. Tiefenbacher (GmbH & Co. KG), Hamburg, Germany; 2 3S Pharmacological Consultation & Research GmbH, Wildeshausen, Germany; 3 Medical-Sanitary Public Institution “Clinical Hospital of the Ministry of Health”, Chisinau, Moldova; 4 Pharma Serv International SRL, Bucharest, Romania

**Keywords:** bioavailability, bioequivalence, dimethyl fumarate, food effect, multiple sclerosis, pharmacokinetics, tegomil fumarate

## Abstract

**Objective:**

Tegomil fumarate is a newly approved treatment for relapsing–remitting multiple sclerosis. Two phase I studies were performed under different fed conditions to evaluate the bioequivalence and tolerability of tegomil fumarate versus dimethyl fumarate in healthy volunteers.

**Methods:**

These were single-center, open-label, randomized, crossover (full replicate design), phase I studies. In both studies, healthy individuals aged 18–55 years received a single dose of tegomil fumarate (348.4 mg gastro-resistant hard capsule; “test”) or an equimolar dose of dimethyl fumarate based on monomethyl fumarate (MMF) release (240.0 mg gastro-resistant hard capsule; “reference”). Participants were randomized to one of two treatment sequences (test–reference–test–reference or reference–test–reference–test), with 7 days between each drug administration. Before each drug administration, participants in Study 1 consumed a low-fat, low-calorie meal (“low-fed”), while those in Study 2 consumed a high-fat, high-calorie meal (“fed”). Pharmacokinetic parameters for MMF (active metabolite in both drugs) and adverse events (AEs) were assessed.

**Results:**

In total, 70 participants were enrolled (Study 1, *N* = 40; Study 2, *N* = 30). In both studies, pharmacokinetic parameters for MMF were similar between drugs. Geometric least-squares mean test/reference ratios for C_max_, AUC_0–t_, and AUC_0–inf_ (and associated 90% confidence intervals for each) all fell within the accepted range of 80.00–125.00%, thereby demonstrating the bioequivalence of tegomil fumarate versus dimethyl fumarate. Both drugs were well tolerated, with no unexpected or serious AEs reported.

**Conclusion:**

These studies demonstrated the tolerability of tegomil fumarate in healthy volunteers. Pharmacokinetic analyses showed that a single dose of 348.4 mg tegomil fumarate released the same amount of MMF as an equimolar dose of 240 mg dimethyl fumarate under both low-fed (low-calorie, low-fat) and fed (high-calorie, high-fat) dietary conditions.

**Clinical trial registration:**

Agenţia Medicamentului şi Dispozitivelor Medicale [AMDM]; https://amdm.gov.md/ro, MMF-BEFI-07-TFB/24 (Rg04-000227) (Study 1; registered September 4, 2024); MMF-BEFI-06-TFB/22 (Rg04-000165) (Study 2; registered July 10, 2023).

## 1 Introduction

Fumaric acid esters have been used in the treatment of psoriasis since the late 1950s, and were first explored as a treatment for multiple sclerosis (MS) about 20 years ago (1, 2). Since then, oral formulations of dimethyl fumarate (Tecfidera®; Biogen Inc.; Cambridge, MA, United States) and diroximel fumarate (Vumerity®; Biogen Inc.; Cambridge, MA, United States) have been approved in the United States and Europe for the treatment of relapsing–remitting MS (3–6). After absorption, dimethyl fumarate and diroximel fumarate are metabolized to monomethyl fumarate (MMF), which is the active moiety responsible for the efficacy of both agents. The pharmacodynamics of MMF are primarily mediated through activation of the nuclear factor (erythroid-derived 2)–like 2 (Nrf2) transcriptional pathway (7–9). Through this pathway, MMF exerts antioxidative and anti-inflammatory effects that may contribute to its cytoprotective properties (9–12). Adverse events (AEs) commonly reported with MMF are flushing and gastrointestinal events (i.e., diarrhea, nausea, abdominal pain, and upper abdominal pain) (4, 6, 13).

Tegomil fumarate (Riulvy®; Neuraxpharm Pharmaceuticals, S. L.; Barcelona, Spain) is a new pegylated, prodrug formulation of MMF, in which two MMF moieties are linked via tetraethylene glycol, a low molecular weight polyethylene glycol (13, 14). After oral absorption of tegomil fumarate, cleavage of the tetraethylene glycol linker releases two clinically active MMF moieties and a pharmacologically inactive polyethylene glycol. Unlike dimethyl fumarate and diroximel fumarate, tegomil fumarate does not produce methanol, a metabolite commonly attributed to gastrointestinal AEs. Therefore, tegomil fumarate may represent a more favorable treatment option for MS with improved gastrointestinal tolerability. Indeed, a randomized, double-blind, phase I study indicated that tegomil fumarate had better gastrointestinal tolerability than dimethyl fumarate in terms of some, although not all, participant-reported assessments (14).

Here, we report the findings from two phase I, single-dose studies, which were conducted to assess the bioequivalence and tolerability of tegomil fumarate (348.4 mg gastro-resistant hard capsules) relative to an equimolar dose of dimethyl fumarate (240.0 mg gastro-resistant hard capsules) in healthy volunteers. Since the approved labels for tegomil fumarate and dimethyl fumarate recommend that each should be administered with food (4, 13), the present studies evaluated bioequivalence under both low-fat, low-calorie (“low-fed”; Study 1) and high-fat, high-calorie (“fed”; Study 2) dietary conditions.

## 2 Materials and methods

### 2.1 Study designs

Both studies were open-label, four-period, two-sequence, crossover (full replicate design), randomized, controlled, single-dose, phase I trials. The studies were conducted at the Medical-Sanitary Public Institution “Clinical Hospital of the Ministry of Health” in Chisinau, Republic of Moldova, between October and December 2024 (Study 1) and between November and December 2023 (Study 2).

Both studies were conducted in agreement with the Declaration of Helsinki and relevant guidelines on Good Clinical Practice and applicable regulatory requirements for bioequivalence studies. Protocols for each study were approved by relevant national ethics committees (Study 1: approval number 1739; Study 2: approval number 1502) and regulatory agencies (Study 1: approval numbers Rg02-004426, Rg04-000227; Study 2: approval numbers Rg02-002628, Rg04-000165), and all study participants provided written informed consent to participate. The protocols and the statistical analysis plans are not publicly accessible; however, data may be made available from the corresponding author upon reasonable request and with the permission of the sponsor.

### 2.2 Study populations

Healthy males and females aged 18–55 years with a body mass index of 18.5–30.0 kg/m^2^ were eligible to participate in these studies. Individuals with a history of hypersensitivity or idiosyncratic reactions to dimethyl fumarate, MMF, or any other fumaric acid ester or polyethylene glycol formulation were excluded. A full list of study inclusion and exclusion criteria for both studies is provided in Supplementary Table S1.

### 2.3 Study procedures

Participants were hospitalized from 6:00 p.m. the evening before study drug administration until 24 h post-dose. All study participants received a standard light dinner that ended ≥10 h before study drug administration (i.e., the evening before). At 30 min before drug administration on study treatment days, participants received either a standard low-fat (~13%), low-calorie (~277.5 kcal) breakfast (Study 1) or a standard high-fat (~57%), high-calorie (~931.4 kcal) breakfast (Study 2), followed by standardized meals at 4, 6, 8, and 12 h post-dose. No other food was consumed; participants could consume water until 1 h before and from 2 h after study drug administration.

Participants received one tegomil fumarate 348.4 mg gastro-resistant hard capsule (“test”; AET Laboratories Private Limited, Gaddapotharam, India; batch number EK148 [Study 1 and Study 2]) or one equimolar dose (based on MMF release) of dimethyl fumarate 240.0 mg gastro-resistant hard capsule (“reference”; Tecfidera, Biogen Netherlands B. V., Badhoevedorp, Netherlands; batch number MFLSA00 [Study 1], KILS081 [Study 2]). Each dose of test and reference medication was initially kept in its original primary package and subsequently placed in an individual secondary package. Participants were assigned a study identification number at random, and the study medication was administered in accordance with the randomization list, with each participant receiving both drugs in sequence across four administration periods, either as test–reference–test–reference or reference–test–reference–test, with a washout of 7 days between administration periods. The terminal half-life of MMF is short (approximately 1 h) (3); thus, a washout period of at least 7 days was considered sufficient to exclude the possibility of any carry-over effect. Randomization was performed by 3S Pharmacological Consultation and Research GmbH. In both studies, the randomization was made in blocks of four subjects using SAS® version 9.4 (SAS Institute Inc., Cary, NC, United States); no other stratification was used. The personnel who enrolled and those who assigned the subjects had access to the randomization plan, since the studies were of an open-label design.

Each capsule was orally administered with 200 mL of still bottled water at room temperature, between 6:00 a.m. and 10:00 a.m. on each study day. Each capsule was swallowed whole, without being crushed, divided, dissolved, sucked, or chewed. Participants were kept in a standing position during drug administration, after which they stayed horizontal or semi-reclined for the next 6 h post-dose.

### 2.4 Sample collection

Within each administration period, blood samples were collected for MMF quantification pre-dose and over 14 h post-dose (Study 1, 25 blood samples in total; Study 2, 24 blood samples in total). All blood samples were stored in an ice-water bath from collection until freezing of plasma samples. Each blood sample was centrifuged to separate the plasma, which was collected in labeled, bar-coded test tubes and stored at ≤ − 20 °C within 60 min of collection. MMF identification and quantification (lower limit of quantification, 6 ng/mL) was conducted by high-performance liquid chromatography–tandem mass spectrometry.

### 2.5 Outcome measures

The following primary pharmacokinetic parameters were calculated for each drug: peak plasma concentration (C_max_); area under the plasma concentration–time curve (AUC) from time zero to the last quantifiable sample (AUC_0–t_); and AUC from time zero to infinity (AUC_0–inf_). Time to C_max_ (T_max_), extrapolated AUC (calculated as [(AUC_0–inf_ − AUC_0–t_)/AUC_0–inf_] × 100%), plasma half-life (t_½_), elimination rate constant (k_el_), and mean residence time (MRT) were also assessed. AEs were monitored throughout the study and coded using Medical Dictionary for Regulatory Activities thesaurus terms (Study 1, MedDRA version 27.0 or later; Study 2, MedDRA version 24.1 or later).

### 2.6 Statistical analyses

The planned study enrolment was 40 participants in Study 1 and 30 participants in Study 2. These sample sizes were estimated to provide 80% statistical power for the primary pharmacokinetic metric (C_max_), based on a significance level (alpha) of 0.05 and an intrasubject coefficient of variation [CV] for C_max_ of above 30%. A potential dropout rate of ≤15% (Study 1) or ≤20% (Study 2) was also taken into consideration.

Descriptive statistics were used to summarize pharmacokinetic metrics (arithmetic mean, geometric mean, standard deviation [SD], CV, median, and range) and AEs (number and proportion of participants). For pharmacokinetic analyses, bioequivalence comparisons were conducted on C_max_, AUC_0–t_, and AUC_0–inf_ for MMF using a general linear model with fixed effects. For these metrics, 90% confidence intervals (CIs) for the ratios of the population geometric least-squares mean (LSM) test/reference ratios were calculated (see Supplementary methods). The standard bioequivalence acceptance range of 80.00–125.00% (for 90% CIs of geometric LSM test/reference ratios) was used for AUC_0–t_ and AUC_0–inf_, while for C_max_, the acceptance range could be widened based on intrasubject variability for the reference product up to a maximum of 69.84–143.19%. T_max_ values (untransformed) were compared using a non-parametric Wilcoxon signed-rank test, with statistical significance defined as *p* < 0.05. Any missing samples were reported as “M.” Non-reportable concentration values, due to no sample remaining for repeat analysis or any other reason as per the in-house procedure, were reported as “NR” and their concentration values were not included for pharmacokinetic and statistical analysis. For AEs, a single sample proportion test was applied (using NCSS software) to investigate differences in the incidence of AEs and differences in the number of study participants having experienced AEs, between the two treatments.

Statistical analyses were performed using SAS® version 9.4 or higher (SAS Institute Inc., Cary, NC, United States).

## 3 Results

### 3.1 Baseline demographics

In Study 1, 65 healthy volunteers were screened and 40 were enrolled, all of whom completed the study and underwent the follow-up examination (Figure 1). The Study 1 population had a mean ± SD age of 37.3 ± 11.8 years, a mean ± SD body mass index of 25.3 ± 3.1 kg/m^2^, and 22 participants (55%) were female (Table 1).

**Figure 1:**
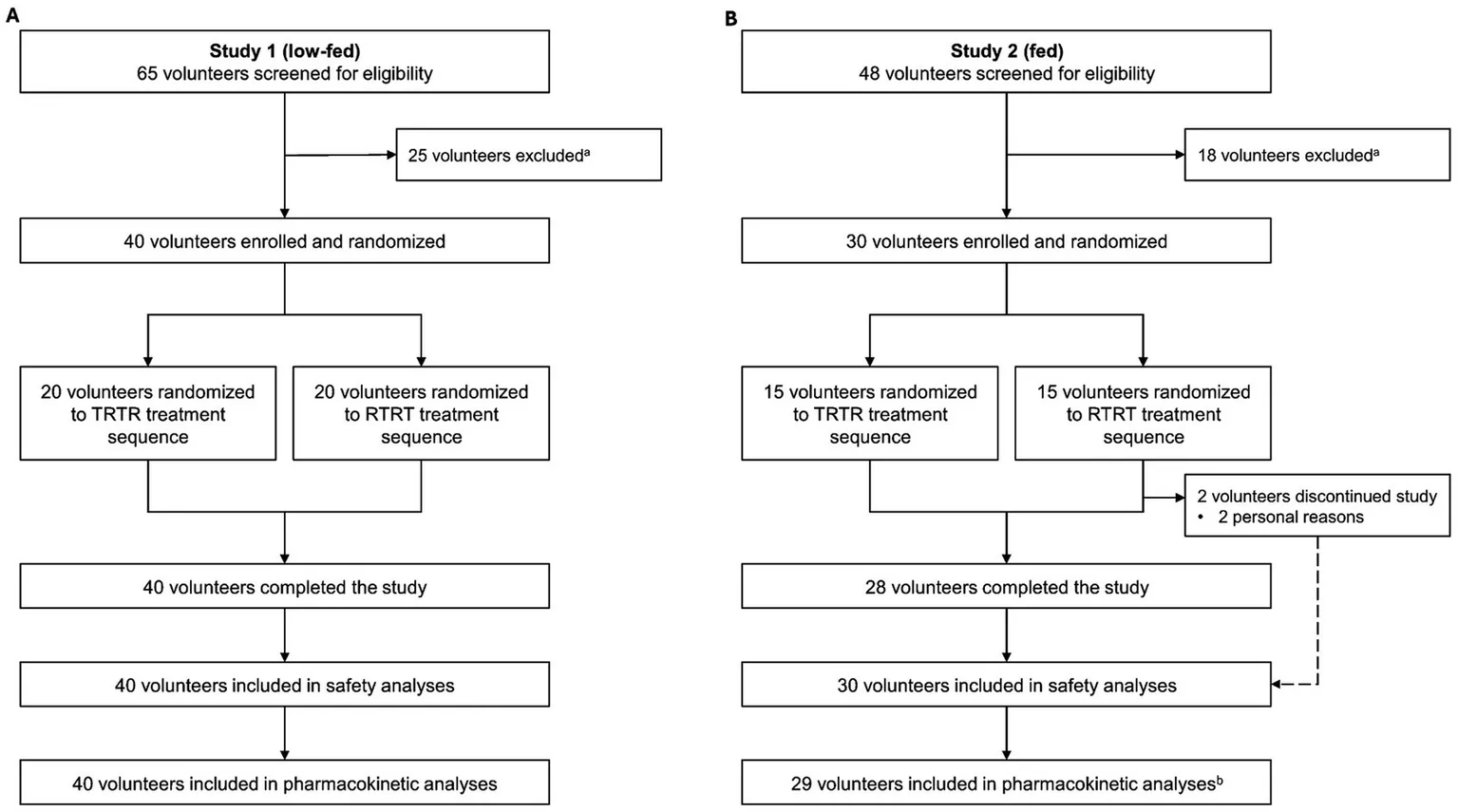
Participant disposition in **(A)** Study 1 and **(B)** Study 2. ^a^The majority of screening failures were due to not meeting inclusion criteria or clinically relevant laboratory abnormalities. ^b^One volunteer who discontinued the study had sufficient data prior to discontinuation for inclusion in pharmacokinetic analyses. RTRT, reference–test–reference–test; TRTR, test–reference–test–reference.

**Table 1:** Baseline characteristics.

	Study 1 (low-fed)	Study 2 (fed)
	*N* = 40	*N* = 30
Sex, n (%)		
Male	18 (45.0)	21 (70.0)
Female	22 (55.0)	9 (30.0)
Age (years), mean ± SD	37.3 ± 11.8	32.0 ± 11.2
Body weight (kg), mean ± SD	72.7 ± 10.0	71.6 ± 10.1
Height (cm), mean ± SD	169.8 ± 8.8	170.5 ± 8.9
BMI (kg/m^2^), mean ± SD	25.3 ± 3.1	24.7 ± 3.2

BMI, body mass index; SD, standard deviation.

In Study 2, 48 healthy volunteers were screened and 30 were enrolled (Figure 1). Of these, 28 participants completed the study, but all 30 underwent the follow-up examination and 29 had data available for the statistical evaluation of pharmacokinetic parameters. The Study 2 population had a mean ± SD age of 32.0 ± 11.2 years, a mean ± SD body mass index of 24.7 ± 3.2 kg/m^2^, and nine participants (30%) were female (Table 1).

### 3.2 Pharmacokinetic parameters

Mean MMF plasma concentration–time profiles for tegomil fumarate and an equimolar dose of dimethyl fumarate (based on MMF release) are shown in Figure 2. Both studies showed no major differences between tegomil fumarate and dimethyl fumarate for the primary parameters of C_max_, AUC_0-t_, and AUC_0-inf_, nor most other pharmacokinetic parameters for MMF, after single-dose administration of each drug (Table 2). However, T_max_ was significantly shorter for tegomil fumarate compared with dimethyl fumarate in both Study 1 and Study 2 (Table 2). Under low-fed conditions (Study 1), the mean (geometric mean) C_max_ value for MMF was 2016.8 (1891.5) ng/mL after tegomil fumarate administration and 1910.7 (1785.1) ng/mL after dimethyl fumarate administration; corresponding values under fed conditions (Study 2) were 1443.5 (1295.6) ng/mL and 1588.0 (1439.2) ng/mL, respectively.

**Figure 2:**
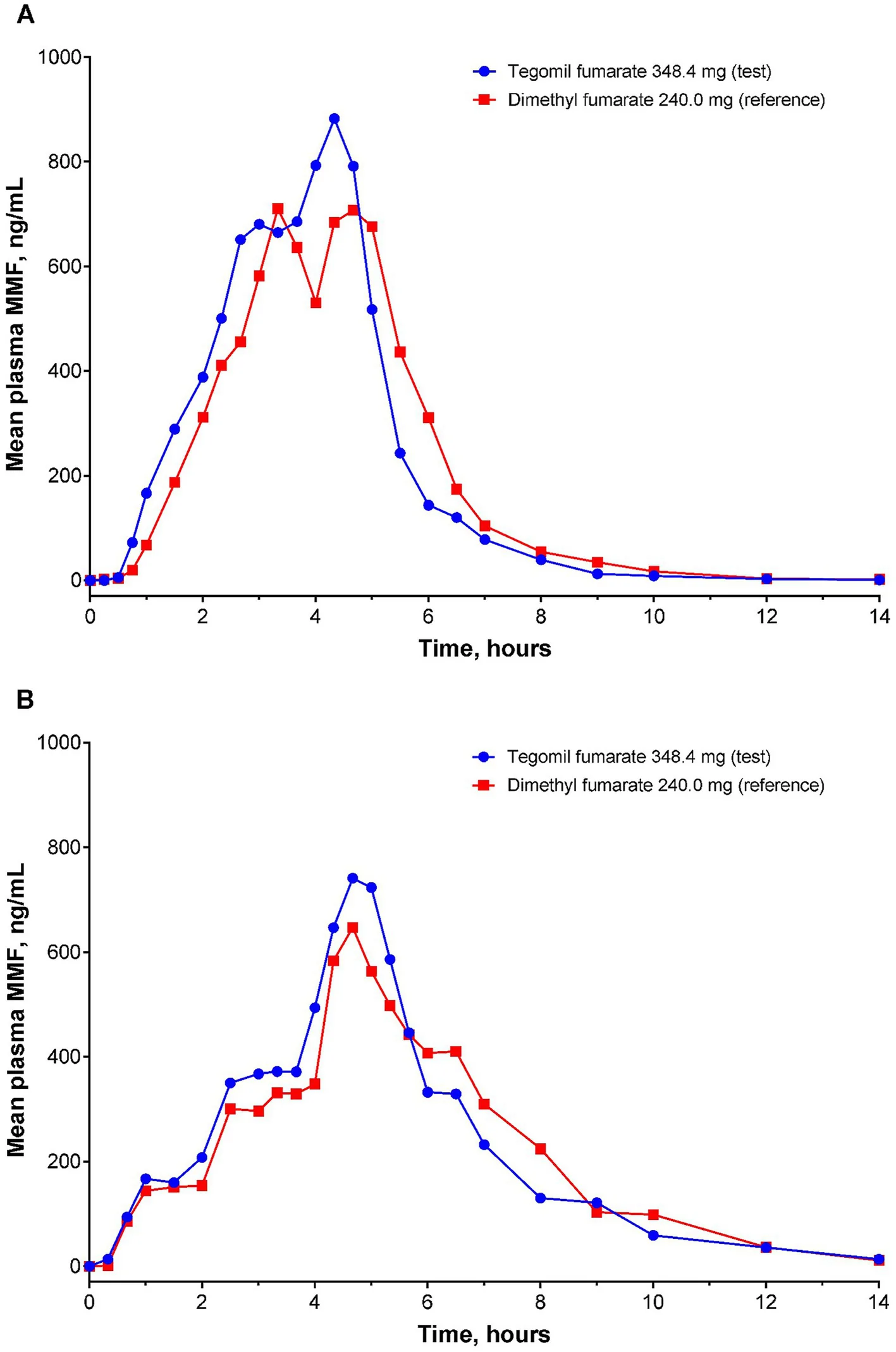
Mean plasma concentration–time curves for MMF after single-dose administration of tegomil fumarate and dimethyl fumarate in healthy volunteers under **(A)** low-fed (Study 1) and **(B)** fed (Study 2) dietary conditions. MMF, monomethyl fumarate.

**Table 2:** Pharmacokinetic parameters for monomethyl fumarate after single-dose administration of tegomil fumarate and dimethyl fumarate in healthy volunteers.

Mean (geometric mean) [SD, CV]^a^	Study 1 (low-fed) *N* exposures = 80^b^		Study 2 (fed) *N* exposures = 58 for reference; 57 for test^b^	
	Tegomil fumarate 348.4 mg (test)	Dimethyl fumarate 240.0 mg (reference)	Tegomil fumarate 348.4 mg (test)	Dimethyl fumarate 240.0 mg (reference)
C_max_, ng/mL	2016.8 (1891.5) [642.0, 31.8]	1910.7 (1785.1) [667.7, 34.9]	1443.5 (1295.6) [673.6, 46.7]	1588.0 (1439.2) [750.5, 47.3]
AUC_0–t_, ng/mL × h	2863.4 (2733.2) [810.8, 28.3]	2765.2 (2665.3) [749.2, 27.1]	2967.6 (2879.0) [728.6, 24.6]	2917.6 (2843.7) [662.9, 22.7]
AUC_0–inf_, ng/mL × h	2887.5 (2758.4) [812.3, 28.1]	2799.3 (2695.3) [774.4, 27.7]	3086.6 (2994.0) [760.1, 24.6]	2981.3 (2904.6) [679.4, 22.8]
Extrapolated AUC, %	0.9 (0.5) [1.7, 188.1]	1.1 (0.4) [2.7, 257.0]	3.6 (1.2) [6.8, 192.3]	2.0 (0.8) [3.5, 172.4]
t_½_, h	1.0 (0.8) [1.0, 102.5]	1.0 (0.7) [1.4, 138.7]	2.0 (1.3) [2.2, 112.6]	1.4 (1.0) [1.4, 102.1]
k_el_, 1/h	1.0 (0.9) [0.4, 41.3]	1.1 (1.0) [0.4, 39.8]	0.7 (0.5) [0.5, 68.5]	0.9 (0.7) [0.5, 54.3]
MRT, h	3.9 (3.7) [1.3, 32.6]	4.3 (4.1) [1.5, 34.2]	5.7 (5.1) [2.5, 43.9]	5.6 (5.2) [2.0, 35.4]
T_max_ (h), median (range)	3.3 (0.7–8.0)*	3.7 (1.0–7.0)	4.7 (0.7–9.0)**	4.7 (0.7–10.0)

^*^p = 0.0159 vs dimethyl fumarate. ^**^p = 0.0495 vs dimethyl fumarate. ^a^Unless otherwise stated. ^b^Both studies were conducted with a 4-period full replicate design, such that both treatments were given twice in each study. AUC, area under the plasma concentration–time curve; AUC_0–inf_, AUC from time zero to infinity; AUC_0–t_, AUC from time zero until the last quantifiable sample; C_max_, peak plasma concentration; CV, coefficient of variation; k_el_, elimination rate constant; MRT, mean residence time; SD, standard deviation; t_½_, plasma half-life; T_max_, time to C_max_.

In Study 1, the mean (geometric mean) AUC_0–t_ for MMF was 2863.4 (2733.2) ng/mL × h after tegomil fumarate administration and 2765.2 (2665.3) ng/mL × h after dimethyl fumarate administration (Table 2). Corresponding values in Study 2 were 2967.6 (2879.0) ng/mL × h and 2917.6 (2843.7) ng/mL × h, respectively. Similarly, the mean (geometric mean) AUC_0–inf_ for MMF was 2887.5 (2758.4) ng/mL × h after tegomil fumarate and 2799.3 (2695.3) ng/mL × h after dimethyl fumarate in Study 1, and 3086.6 (2994.0) and 2981.3 (2904.6) ng/mL × h, respectively, in Study 2.

Analysis of variance showed no significant effect of sequence, period, or treatment on the primary pharmacokinetic parameters in either study. A subject-within-sequence effect was observed on all three primary parameters in both studies, reflecting the usual inter-individual variations.

### 3.3 Bioequivalence comparison

Geometric LSM test/reference ratios for C_max_, AUC_0–t_, and AUC_0–inf_ are presented in Table 3. Since the 90% CI for each geometric LSM test/reference ratio was within the accepted range (80.00–125.00%) in both studies, the widening of acceptance range for C_max_ was not invoked, and the bioequivalence of tegomil fumarate and an equimolar dose of dimethyl fumarate—with respect to rate and extent of MMF absorption—was established under both low-fed and fed conditions.

**Table 3:** Bioequivalence comparison of primary pharmacokinetic parameters for monomethyl fumarate after single-dose administration of tegomil fumarate (test) and dimethyl fumarate (reference) in healthy volunteers.

Geometric LSM test/reference ratio (90% CI)	Study 1 (low-fed) *N* = 40 subjects^a^	Study 2 (fed) *N* = 29 subjects^a^
C_max_	105.96 (96.55, 116.29)	89.38 (80.03, 99.81)
AUC_0–t_	102.55 (98.48, 106.79)	101.22 (96.67, 105.98)
AUC_0–inf_	102.34 (98.31,106.54)	103.02 (98.46, 107.78)

^a^Both studies were conducted with a 4-period full replicate design, such that both treatments were given twice in each study. AUC, area under the plasma concentration–time curve; AUC_0–inf_, AUC from time zero to infinity; AUC_0–t_, AUC from time zero until the last quantifiable sample; CI, confidence interval; C_max_, peak plasma concentration; LSM, least-squares mean.

### 3.4 Adverse events

In Study 1, 58 AEs were reported in 25 participants, and all were either mild (69.0% of AEs) or moderate (31.0%) in severity. AEs were reported after administration of tegomil fumarate in 21 participants (32/58 AEs [55.2%]) and after dimethyl fumarate in 18 participants (26/58 AEs [44.8%]). In Study 2, 83 AEs were reported in 25 participants; 81.9% of AEs were mild and 18.1% were moderate in severity. These AEs emerged after administration of tegomil fumarate in 17 participants (32/83 AEs [39.8%]) and after dimethyl fumarate in 23 participants (51/83 AEs [61.0%]).

In both studies, there were no severe or serious AEs, and no statistically significant differences between drugs were noted for the incidence of AEs. All study participants who experienced AEs recovered completely before study end, and no clinically significant changes occurred in vital signs or laboratory parameters.

In Study 1, the most frequent treatment-related AEs were skin hyperemia, pricking skin sensation, and feeling hot; in Study 2, the most frequent treatment-related AEs were skin hyperemia, feeling hot, and itching (Table 4). In both studies, the incidence of treatment-related AEs after tegomil fumarate was comparable with the incidence of treatment-related AEs after dimethyl fumarate.

**Table 4:** Incidence of treatment-related adverse events.

Treatment-related AEs, %^a^	Study 1 (low-fed) *N* = 40		Study 2 (fed) *N* = 30	
	Tegomil fumarate	Dimethyl fumarate	Tegomil fumarate	Dimethyl fumarate
Skin hyperemia	33.8	25.0	26.3	42.4
Pricking skin sensation	5.0	7.5	1.8	6.8
Feeling hot	1.3	0.0	12.3	15.3
Itching	0.0	0.0	7.0	6.8
Nausea	0.0	0.0	1.8	1.7

^a^Number of events/total number of drug exposures, expressed as percentage. In Study 1, there were 80 drug exposures each for tegomil fumarate and dimethyl fumarate; in Study 2, there were 59 drug exposures for tegomil fumarate and 57 for dimethyl fumarate. AEs, adverse events.

## 4 Discussion

These two phase I studies revealed no major differences in the primary pharmacokinetic parameters for MMF (i.e., C_max_, AUC_0–t_, AUC_0–inf_) after single-dose administration of tegomil fumarate versus an equimolar dose of dimethyl fumarate (based on MMF release) in healthy volunteers. Under both low-fed (low-fat, low-calorie) and fed (high-fat, high-calorie) dietary conditions, these two formulations were bioequivalent, as the 90% CIs for geometric LSM test/reference ratios for the primary pharmacokinetic parameters were all within the accepted range of 80.00–125.00%.

These findings are clinically important, as they clearly demonstrate the bioequivalence of tegomil fumarate (first approved in Europe in July 2025 (13)) and a traditional oral formulation of dimethyl fumarate (first approved in Europe in January 2014 (4)). Moreover, we demonstrated bioequivalence under both low-fat, low-calorie (Study 1) and high-fat, high-calorie (Study 2) conditions. Given that tegomil fumarate and dimethyl fumarate should both be taken with food (4, 13), data from these studies suggest that the size and the composition of the meal have no impact on the bioavailability of tegomil fumarate relative to dimethyl fumarate.

Epidemiological trends highlight an increasing incidence and disease burden of MS, especially in Western Europe (15). Robust long-term data (e.g., DEFINE, CONFIRM, ENDORSE) have confirmed the marked clinical efficacy of dimethyl fumarate in patients with relapsing–remitting MS; however, gastrointestinal AEs are common (16–19). Tegomil fumarate was developed as an alternative fumaric acid ester with improved gastrointestinal tolerability; indeed, a recent randomized phase I study found that the incidence, severity, and duration of gastrointestinal AEs were lower among healthy volunteers receiving tegomil fumarate versus dimethyl fumarate, with some (although not all) participant-reported assessments favoring tegomil fumarate (14). In the current studies, single doses of tegomil fumarate and dimethyl fumarate were generally well tolerated in healthy individuals; no unexpected or serious AEs were reported, all AEs were mild or moderate and had resolved before study end, and there were no significant differences in AE incidence between the two treatments. However, it should be noted that, given the single-dose design, this study was not intended to provide a comprehensive comparison of the safety and tolerability profiles of the two products.

There are potential limitations that should be considered when interpreting the results of these studies. These limitations included those typically associated with phase I trials, such as small sample size and short duration, as well as the possibility that results may not be fully generalizable to larger, more varied populations. However, these potential restrictions are unlikely to markedly influence the impact of our findings because the studies were well-designed and conducted, and were planned with sufficient statistical power to clearly confirm bioequivalence of the two products evaluated (20). It is acknowledged that the studies were not conducted concurrently and included demographically distinct cohorts; as such, comparison of results of the two prandial conditions should be made with caution.

In conclusion, in healthy adults, single-dose administration of tegomil fumarate gastro-resistant hard capsules was bioequivalent to an equimolar dose (based on MMF release) of dimethyl fumarate gastro-resistant hard capsules with respect to both the rate (i.e., C_max_) and extent (i.e., AUC_0–t_, AUC_0–inf_) of systemic exposure to MMF. Bioequivalence was established under two distinct prandial conditions—after a low-fat, low-calorie meal and after a high-fat, high-calorie meal—with the 90% CIs of the geometric LSM test/reference ratios for C_max_, AUC_0–t_, and AUC_0–inf_ contained within the conventional 80.00–125.00% acceptance interval in both states. Following single-dose administration, the incidence of adverse events was comparable between tegomil fumarate and dimethyl fumarate gastro-resistant hard capsules, with no statistically significant differences detected. The potentially improved gastrointestinal tolerability of tegomil fumarate relative to dimethyl fumarate was indicated in a previous report (14). The present pharmacokinetic findings indicate that tegomil fumarate may be administered irrespective of meal size or caloric and fat content, without clinically significant changes in systemic exposure to MMF. From a patient-management perspective, this indicates dosing flexibility and suggests that patients may be able to take tegomil fumarate with either a large meal or a light snack. Taken together, these data support tegomil fumarate gastro-resistant hard capsules as a pharmacokinetically robust treatment option for patients with relapsing–remitting MS.

## Data Availability

The raw data supporting the conclusions of this article will be made available by the authors, without undue reservation.
