# Supplementary material for: Bioequivalence of tegomil fumarate gastro-resistant capsules versus dimethyl fumarate gastro-resistant capsules under different fed conditions: two randomized, crossover, phase I studies
Source: Front Neurol. 2026 Sep 29;17:1936043. doi: 10.3389/fneur.2026.1936043 (PMC13657950; doi:10.3389/fneur.2026.1936043)
Supplement: Supplementary file 1 [file Supplementary_file_1.docx]

**Supplementary Material**

**Supplementary methods**

***Statistical analysis***

The bioequivalence comparison was conducted for peak plasma concentration (C_max_), area under the plasma concentration–time curve (AUC) from time zero to the last quantifiable sample (AUC_0–t_), and AUC from time zero to infinity (AUC_0–inf_) for monomethyl fumarate (MMF). For these parameters, a 90% confidence interval (CI) for the ratio of the population geometric least-squares mean (LSM) test/reference ratio was calculated. An initial analysis of variance (ANOVA) was implemented using a general linear model. The ANOVA model used sequence, period, participant within sequence, and treatment as fixed effects; the level of significance was set at 0.1 for the sequence effect and 0.05 for all remaining effects.

To evaluate C_max_ using scaled criteria, intra-individual variability for dimethyl fumarate (reference) was calculated by excluding the tegomil fumarate (test) formulation from the model specifications. The model was applied to natural-log–transformed data considering sequence, participant within sequence, and administration period as fixed effects. Participants considered for this evaluation had to have C_max_ values available for both dimethyl fumarate administration periods. If participants had only one dimethyl fumarate C_max_ value available, they were excluded from the estimation of intra-individual variability, but were included in the 90% CI evaluation.

**Supplementary Table 1.** Eligibility criteria for both studies.

**Inclusion criteria**

1. Healthy adult males and females aged 18–55 years

2. Body mass index 18.5–30.0 kg/m^2^

3. Normal hematology, clinical chemistry, and urinalysis (some deviations outside normal limits may be acceptable, if considered not clinically significant by the Investigator)

4. No renal or hepatic impairment

5. No cardiovascular or respiratory diseases

6. No acute or chronic infectious disease

7. Non-smokers, or ex-smokers who gave up smoking ≥2 years before the study

8. Participants agree to abstain from alcohol for 48 hours before, and 24 hours after, study drug administration

9. Participants agree to abstain from beverages or food containing methylxanthines (coffee, tea, cola, energy drinks, chocolate, etc.), St John’s Wort, and chewing gum for 48 hours before, and 24 hours after, study drug administration

10. Participants agree to abstain from beverages or food containing grapefruit or oranges for 72 hours before, and 24 hours after, study drug administration

11. Participants understand the full nature and purpose of the study, including possible risks and side effects; and are able to cooperate with the Clinical Investigator and to comply with all study requirements

12. Informed written consent provided before the start of study screening

13. For female participants only: not pregnant or lactating, and agree to use an effective non-hormonal contraceptive method or abstain from sexual intercourse from the start of screening until the follow-up examination or 3 weeks post-dose, whichever is longest

14. Participants, who in the opinion of the Clinical Investigator, are medically fit for participation in the study

15. Negative COVID-19 test result at screening (Study 2 only)

**Exclusion criteria**

1. History of hypersensitivity or idiosyncratic reactions to dimethyl fumarate, monomethyl fumarate, or any other fumaric acid ester, or polyethylene glycol formulation

2. Participation in a clinical study with a new investigational product in the preceding 3 months, or with a generic product in the preceding 2 months

3. Participants intending to be hospitalized within 3 months after study drug administration

4. Hospitalization for any reason within the 8 weeks before study start

5. Donation of ≥450 mL of blood within the 8 weeks before study start

6. Intake of any prescribed or non-prescribed drug or vitamin supplement within the 2 weeks before study start

7. Intake of any prescribed or non-prescribed drug that acts as an inhibitor/competitor of the active renal elimination pathway within the 2 weeks before study start

8. History or presence of any relevant medical condition (e.g., cancer, significant disease of the renal, hepatic, gastrointestinal, respiratory, cardiovascular, endocrine, or locomotor systems; or any metabolic, hematological, or neurological disorder)

9. History or actual presence of coagulation disorders such as thromboembolic diseases (e.g., venous thrombophlebitis, pulmonary embolism, or coagulation factor deficiency), cerebrovascular diseases, angina pectoris, myocardial infarction or coronary arterial disease, porphyria

10. History of hemophilia (A, B, or C)

11. Absolute leukocyte or lymphocyte count below the LLN, eosinophils above the ULN, or hemoglobin below the LLN, at screening

12. Any elevation of liver function tests (alanine aminotransferase, aspartate aminotransferase, γ-glutamyl transpeptidase, bilirubin, or alkaline phosphatase above the ULN) at screening

13. Proteinuria (more than trace), including elevated blood urea nitrogen, at screening

14. Serum creatinine >1.5 × ULN at screening

15. Reported history of, or current treatment for, gastrointestinal disease such as diverticulitis, diverticulosis, irritable bowel syndrome, ulcer, inflammatory bowel disease (Crohn’s or ulcerative colitis), upper gastrointestinal disorders (such as dyspepsia, heartburn), gastroesophageal reflux esophagitis, or history of significant abdominal surgeries including cholecystectomy and/or appendectomy, or conditions (such as abdominal gunshot wounds)

16. History of gastrointestinal bleeding or perforation; history of partial or total gastrectomy

17. History of achlorhydria or surgery bypassing or excluding the duodenum

18. History of significant gallbladder/biliary tract disease, liver tumors, or any liver function disorders

19. History of bladder outlet obstruction

20. History of a medically significant past or present recurrent medical condition, generally and/or frequently associated with gastrointestinal events such as nausea, vomiting, constipation, abdominal pain, and diarrhea

21. History of medically significant flushing symptoms (e.g., flushing reaction to food)

22. History of seizures, other than febrile seizures as a child

23. Participants with heart rate outside the normal range of 50–100 bpm, body temperature outside the normal range of 35.5–37.4°C, respiratory rate outside the normal range of 14–20 breaths per minute, or sitting blood pressure <90/60 mmHg or >140/90 mmHg, at screening

24. Electrocardiogram evidence of clinically significant abnormalities

25. Active virus infection within the 4 weeks before study start

26. Immunization within 2 weeks before study start

27. Any recent (within the last 2 years) history of drug or alcohol abuse, recent psychiatric disorder, or use of psychotropic medicines

28. Any current condition or other disease known to interfere with the absorption, distribution, metabolism, or excretion of medicines

29. Participants following particular/specified diets for any reason

30. Positive test result for HIV, hepatitis C, or hepatitis B

31. A history of difficulty in donating blood

32. Participants known or likely to be non-compliant, unreliable, or incapable of understanding the information related to risks and discomfort; or in a precarious financial situation that may influence the decision to participate

bpm, beats per minute; HIV, human immunodeficiency virus; LLN, lower limit of normal; ULN, upper limit of normal.
